# Beyond Metformin: A Review of Non-pharmacological Treatments for Polycystic Ovary Syndrome

**DOI:** 10.7759/cureus.103619

**Published:** 2026-02-14

**Authors:** P. Indira Lakshmi, Sujatha KJ, Prashanth Shetty

**Affiliations:** 1 Clinical Naturopathy, Sri Dharmasthala Manjunatheshwara (SDM) College of Naturopathy and Yogic Sciences, Ujire, IND; 2 Natural Therapeutics, Sri Dharmasthala Manjunatheshwara (SDM) College of Naturopathy and Yogic Sciences, Ujire, IND; 3 Yoga, Sri Dharmasthala Manjunatheshwara (SDM) College of Naturopathy and Yogic Sciences, Ujire, IND

**Keywords:** complementary and alternative medicine, hydrotherapy, metformin, naturopathy, polycystic ovary syndrome

## Abstract

Polycystic ovary syndrome (PCOS) affects reproductive-aged women through hereditary, biological, environmental, and lifestyle factors including stress, poor diet, and oxidative stress. This narrative review aims to systematically synthesize evidence on non-pharmacological naturopathic treatments (yoga, hydrotherapy, diet, and acupuncture) for improving insulin sensitivity, reducing obesity, and alleviating PCOS symptoms. While conventional treatments like metformin increase insulin sensitivity and clomiphene promote ovulation, they fail to address underlying causes and carry side effects, necessitating complementary approaches. The objective of this review is to investigate databases, including Web of Science, PubMed/MEDLINE, and Scopus were searched from October 1, 1998, to January 16, 2025, using Boolean combinations of keywords such as “PCOS,” “naturopathy,” “complementary and alternative medicine,” “chromotherapy,” “metformin,” “insulin sensitivity,” “obesity,” and “diabetes.” Naturopathic interventions consistently demonstrated favorable effects on PCOS outcomes across reviewed studies. Yoga therapies improved insulin sensitivity, reduced inflammatory responses, and enhanced quality of life, with only minimal adverse effects reported. Hydrotherapy and dietary approaches effectively addressed obesity parameters and PCOS clinical symptoms. Combined non-pharmacological modalities showed synergistic benefits over pharmacological treatments alone, characterized by high patient adherence and excellent safety profiles. Complementary therapies provide safer ways to manage PCOS compared to using medication alone. Combining these methods needs clinical guidelines and randomized controlled trials.

## Introduction and background

Polycystic​‍​‌‍​‍‌​‍​‌‍​‍‌ ovary syndrome (PCOS) is the most common endocrinopathy that affects women of reproductive age, with a prevalence rate of 8% to 13% that varies depending on the population studied [1]. The symptoms usually start appearing when the patient is between 18 and 39 years old. It is marked by symptoms such as excessive hair growth, acne, high insulin levels, and irregular menstrual cycles [2]. Metabolic problems such as insulin resistance (IR) and obesity, which are common in 60 to 80% of women with PCOS, are reported [3]. Negative mental health effects and reduced quality of life are frequently found in these patients. Up to 20% of women with fertility problems, such as difficulty in conceiving and early pregnancy loss, are the ones that have PCOS [4]. PCOS is a disorder that has a combination of symptoms, mainly those of androgen excess (including hirsutism and/or hyperandrogenemia) and ovarian problems (like oligo-ovulation and/or polycystic ovarian morphology (PCOM)). The Rotterdam criteria define the diagnosis of PCOS as the presence of at least two of the three features [5]. The severity of signs and symptoms can vary greatly between individuals, and the syndrome is currently considered a multisystem disorder with consequences for cardiovascular and metabolic health in the long run [6]. The heredity factor involves those families where there is a history of PCOS among the first-degree relatives. According to the research, 35% of premenopausal women among 93 PCOS patients and 40% of their sisters were affected with this disorder. Genetic factors that lead to PCOS etiology include SNPs (single-nucleotide polymorphisms) in specific genes. There are almost 241 gene variants linked to PCOS. These gene variations can be either polymorphisms or single-nucleotide changes, which can alter a gene's transcriptional activity. These genes are involved in steroidogenesis, ovarian theca cell functioning, and hypothalamic-pituitary hormone secretion [7]. Environmental factors that may contribute to the development of PCOS during childhood are lifestyle, unhealthy eating habits, lack of physical activity, and environmental chemicals [8]. Reactive oxygen species are the main culprit for oxidative damage to DNA and lipid peroxidation. Oxidative damage to DNA is also cancer-causing. Research shows that DNA damage as a result of oxidative stress is associated with ovarian carcinoma in PCOS patients [9]. IR and hyperinsulinemia play a central role in the development of hyperandrogenism, one of the mechanisms by which IR and hyperinsulinemia trigger theca cells in the ovaries to release excess androgens and luteinizing hormone [10]. Obesity is one of the main causes that leads to defective insulin metabolism, thus acting as a factor in the acceleration of diabetes in PCOS. PCOS is characterized by excessive body fat in the abdomen, hips, and thighs, referred to as central adiposity. Abdominal adiposity, thus, is a further stimulant for androgen secretion, hyperadiponectinemia, cytokine secretion, oxidative stress, and hyperinsulinemia [11]. While pharmacological treatments like metformin, oral contraceptives, and clomiphene effectively manage specific PCOS symptoms, their limitations, side effects, poor adherence, and incomplete metabolic resolution, underscore the need for non-pharmacological alternatives. Lifestyle modifications, including sustained weight loss (5-10%) through diet and exercise, remain first-line, complemented by emerging naturopathic therapies such as yoga (stress reduction, insulin sensitivity), hydrotherapy (circulation, detoxification), acupuncture (neuroendocrine balance), and mind-body practices. These modalities target PCOS's multifactorial pathology through holistic mechanisms, including autonomic regulation, anti-inflammatory pathways, adipokine modulation, and improved ovulatory function, warranting systematic evidence synthesis for clinical integration. Given PCOS's complex metabolic challenges and the limitations of pharmacological treatments, naturopathic therapies (yoga, hydrotherapy, and diet) offer holistic alternatives. This narrative review synthesizes their evidence for improving IR, obesity, and symptoms. It aims to guide integrated clinical management strategies.

## Review

Multifactorial pathophysiology of PCOS: role of hyperandrogenism, insulin resistance, inflammation and gut dysbiosis

PCOS is a disorder that has a combination of symptoms, mainly those of androgen excess (including hirsutism and/or hyperandrogenemia) and ovarian problems (like oligo-ovulation and/or PCOM. Androgen‍‌‍‍‌‍‌‍‍‌ excess is regarded by some researchers as the main feature of PCOS; nevertheless, only 80-85% of women with clinical hyperandrogenism are diagnosed with PCOS, and acne and diffuse alopecia are even less specific [12]. During ovarian follicular development, one group of follicles is selected for further growth from the recruited primordial follicles. From this growing follicle, one antral follicle is chosen to ovulate. These processes require the reproductive, metabolic, and intraovarian systems to interact very closely and in a coordinated way. PCOS is a disorder in which ovarian hyperandrogenism, hyperinsulinemia caused by IR, and altered intraovarian paracrine signaling may interfere with follicle growth [13]. Additionally, hyperinsulinemia increases the level of serum free testosterone by decreasing hepatic sex hormone-binding globulin (SHBG) production, increases serum insulin-like growth factor-1 bioactivity by suppressing IGF-binding protein production, and works in tandem with luteinizing hormone (LH) to increase androgen production and bind to IGF-1. Conversely, an excess of insulin causes premature follicle luteinization by promoting granulosa cell differentiation induced by follicle-stimulating hormone, which stops granulosa cell proliferation and subsequent follicle growth [14]. Adipose tissue, which is a major factor in this condition, secretes nearly 100 substances that regulate various functions like metabolism, appetite, neural activity, digestion, and inflammation. Besides this, the tissue is heavily infiltrated by macrophages, and the crosstalk between adipocytes, macrophages, and pluripotent cells leads to complex paracrine interactions. In particular, paracrine dysregulation of adipokine (e.g., adiponectin) production by macrophage-secreted cytokines in PCOS promotes the development of IR [15]. Adipokine release, stromal fat immune cell recruitment, and activation are all associated with chronic stress. In addition, it contributes to the development of an inflammatory condition by raising the levels of inflammatory cytokines like tumor necrosis factor and Interleukin-6 and by upsetting the balance between oxidants and antioxidants [16]. Stress activates the hypothalamic-pituitary-adrenal axis to secrete cortisol. After that, cortisol initiates IR by promoting visceral fat accumulation, gluconeogenesis, and lipolysis. In addition, cortisol stimulates glucose production in the liver. Stress is also involved in the enhancement of insulin levels. Other stress-related influences on PCOS may also point to anti-Mullerian hormone interference and changes in sex hormone levels [17]. Intake of saturated fatty acids plays a role in the development of PCOS by creating an inflammatory status and by lowering insulin sensitivity [18]. Vitamin D deficiency may worsen the case of PCOS or comorbidities caused by PCOS. Calcitriol elevates insulin receptor levels at the messenger ribonucleic acid and protein stages. It also raises insulin sensitivity both directly and indirectly. The direct effect is through the activation of peroxisome proliferator-activated receptor gamma, the receptor involved in fatty acid metabolism in adipose tissue and skeletal muscle. The indirect effect is the regulation of intracellular calcium, which is very important for insulin-mediated signaling in fat and muscle. On the flip side, lack of vitamin D may lead to the development of IR through an inflammatory response. Besides that, vitamin D downregulates the anti-Mullerian hormone promoter [19]. Recently, researchers have highlighted that gut dysbiosis in PCOS might be a significant factor behind obesity, IR, and inflammation. Poor diet quality may facilitate the movement of lipopolysaccharides released by gram-negative bacteria into the bloodstream. This provokes systemic inflammation. PCOS is associated with chronic low-grade inflammation that is characterized by elevated levels of white blood cells, C-reactive protein, interleukin-6, and interleukin-18, as well as inflammatory proteins such as monocyte chemoattractant protein-1 and macrophage inflammatory protein-1. Inflammation is very closely related to IR, and hyperglycemia can cause excessive production of Tumor necrosis factor-α, thus leading to the worsening of PCOS symptoms. Apart from that, advanced glycation end products and their receptors, which are responsible for the promotion of inflammation and oxidative stress, are also highly expressed in women with PCOS [20].

Conventional management

The first-line pharmacological treatment for anovulatory infertility in women with PCOS is clomiphene, which is a selective estrogen receptor modulator, and insulin-sensitizing agents like metformin are used to improve insulin sensitivity, reduce androgen levels, and enhance fertility [21]. For those resistant to oral treatments, gonadotropin injections or surgical options such as laparoscopic ovarian drilling may be considered. Clomiphene responders carry a higher risk of ovarian hyperstimulation and multiple pregnancies [22]. For managing symptoms like hirsutism, acne, and menstrual irregularities, combined oral contraceptive pills are recommended as first-line therapy. These pills increase SHBG and suppress LH, reducing ovarian androgen production and improving symptoms [23].

Mechanism of action of metformin in PCOS

Metformin​‍​‌‍​‍‌​‍​‌‍​‍‌ or dimethyl biguanide is an orally administered antidiabetic drug. Metformin is mainly used to prevent diabetes and also used in the PCOS condition. Metformin is capable of making menstrual cycles more regular and can also enhance fertility [24]. Metformin decreases IR, thereby helping ovulation and menstrual regularity in women with PCOS. It reduces glucose production in the liver, improves insulin sensitivity in muscles, and decreases glucose absorption in the gut, which leads to lower insulin levels and slight weight loss. Metformin at the cellular level imitates energy-deficient situations by inhibiting mitochondrial function; thus, AMPK, a key energy regulator, is activated. AMPK enhances glucose uptake and lessens fat synthesis; thus, triglyceride levels are lowered, and insulin sensitivity is improved [25].

Methodology

The narrative review searched articles from various databases (PubMed/MEDLINE, Scopus, Web of Science) from October 1, 1998, to January 16, 2025, using MeSH terms, Boolean operators, and keywords ("PCOS" OR "polycystic ovary syndrome" OR "naturopathy" OR "complementary and alternative medicine") AND ("hydrotherapy" OR "acupuncture" OR "yoga" OR "aromatherapy" OR "massage" OR "chromotherapy" OR "magnetotherapy" OR "diet" OR "herbal"). It focused on non-pharmacological therapies' effects on hormonal balance, insulin sensitivity, inflammation, metabolic health, and reproductive outcomes in humans, with a review of hydrotherapy, acupuncture, yoga, and diet's impact on hyperandrogenism, IR, menstrual regularity, and psychological well-being.

Non-pharmacological treatment

Complementary and alternative medicine (CAM) encompasses various approaches aimed at enhancing treatment efficacy while minimizing side effects. Key interventions within CAM include Chinese herbal medicine, acupuncture, moxibustion, medicinal foods, yoga, aromatherapy, vitamin therapy, diet therapy, and psychotherapy. Research indicates that the use of CAM strategies can significantly influence hormone levels, reduce IR, facilitate weight loss, improve mood regulation, and enhance ovulation in women suffering from infertility due to PCOS [26]. A 2025 systematic review and network meta-analysis concluded that non-pharmacological interventions, particularly electroacupuncture combined with exercise and dietary management, effectively reduce androgen levels in PCOS patients [27].

Hydrotherapy and PCOS

A​‍​‌‍​‍‌​‍​‌‍​‍‌ cold sitz bath results in active dilation of the vessels in the lower abdomen. The thermic reaction from the bath increases the nutritive processes in the parts concerned, and it excites the contraction of the muscular structures of the viscera; hence, the pelvic organs are influenced along with the various musculoskeletal structures that support the pelvic viscera. A longer cold sitz (15-20 min) is responsible for very marked effects on the pelvic circulation [28]. The involuntary muscles of the uterus, as well as those of other pelvic organs, are excited by very hot (10 min) foot applications. The blood vessels in the feet are dilated by this application, and the resultant dilatation extends not only to the upper parts of the limbs but also to the vessels of the pelvic viscera. The femoral artery after a hot foot bath shows this effect by its vigorous pulsation [29]. Peat substances, when absorbed through the skin during mud pack treatments, result in a long-lasting vasodilation and consequently improve pelvic blood flow. This therapy lowers proinflammatory cytokines such as interleukin-1 and TNF-α, oxidative stress markers, and nitric oxide production, thus reducing systemic inflammation that is associated with PCOS [30]. A cold enema facilitates liver and kidney functions and promotes the cleansing of the alimentary canal. Thus, it indirectly helps hormonal balance and metabolism, which are relevant for PCOS [31].

Acupuncture and PCOS

Acupuncture,​‍​‌‍​‍‌​‍​‌‍​‍‌ one of the complementary and alternative therapies, is receiving attention in the treatment of PCOS patients with menstrual disorders. Various randomized controlled trials (RCTs) and meta-analyses have pointed to the fact that acupuncture might enhance the reproductive outcomes in PCOS through the regulation of the hypothalamic-pituitary-ovarian axis, interaction with the sympathetic nervous system, and by improving IR and endometrial receptivity [32]. Researchers have found that acupuncture not only aids reproductive endocrine function but also lessens ovulatory dysfunction and follicular development disorders by way of inhibiting the PI3K/AKT/mTOR pathway and regulating granulosa cell autophagy via lowering the LncMEG3 expression [33]. Moreover, abdominal acupuncture has significantly enhanced IR in obese-type PCOS patients, which can be attributed to the acupuncture treatment's effect on body mass index, waist-to-hip ratio (WHR), and lipid metabolism dysfunctions [34]. A recent investigation also revealed that EA seems to alleviate anxiety and depression symptoms apart from regulating the serum levels of norepinephrine and serotonin in unmarried PCOS patients [35]. Throughout these literature studies, acupuncture points such as SP6, ST29, CV6, LI4, CV3, ST36, SP9, and CV4 have been frequently mentioned. In reality, the acupoint that has been predominantly put into practice is SP6. As per the traditional Chinese medicine (TCM) theory, SP6 (Sanyinjiao) is mainly known for its functions to nourish black, release blood, calm the liver, and regulate Qi, which would eventually assist in resolving the problems of the female reproductive system. The acupoint that was involved in the second most was ST29 (Guilai), and it was employed extensively to reduce hyperandrogenism and ovulatory dysfunction and also to adjust the menstrual cycle. Treatment with ST29 helps the blood to flow normally by getting rid of the stasis, thus regulating menstruation and easing the pain [36].

Aromatherapy and PCOS

Essential oils are gaining popularity among women with PCOS symptoms compared to traditional treatments involving hormones, lifestyle changes, and surgeries. Some oils reduce inflammation, balance hormones, and support ovarian and uterine health [37]. Essential oils can treat PCOS topically, internally, or by inhalation. Relief can be found by applying a mix of preferred oil and carrier oil on the lower abdomen. Additionally, spearmint oil supports mental health, aiding in PCOS recovery [38].

Massage and PCOS

Massage, a soft tissue manipulation technique utilizing touch, friction, kneading, percussion, and vibration, can be therapeutic when incorporating oils like essential oils. While empirical research on its effectiveness for PCOS is limited, some studies suggest that it can improve sleep quality, reduce inflammatory cytokines, enhance anti-inflammatory pathways, lower IR via the AMPK pathway, and decrease stress hormones. Abdominal massage serves as a non-invasive fertility treatment for PCOS, enhancing blood flow to reduce stagnation, stiffness, and menstrual pain. It lowers stress, WHR, and cyst size while offering a revolutionary alternative to pharmacological or invasive methods. By improving oxygenation to eggs, destroying cysts, and alleviating depression/anxiety, it promotes overall reproductive health [39]. 

Physical activity and PCOS

Managing PCOS effectively involves structured physical activity, which positively influences health outcomes by improving insulin sensitivity through mechanisms such as GLUT4 translocation and AMPK activation in muscles. It also reduces free androgen levels by increasing SHBG and decreasing hyperinsulinemia, leading to better ovarian function, more ovulation, and regular menstruation, primarily linked to changes in body composition rather than direct hormonal effects [40]. Besides that, cardiometabolic advantages of physical activity in PCOS span over slight reductions in waist circumference, better lipid profiles, improved mitochondrial function, and increased cardiorespiratory fitness [41].

Chromotherapy and PCOS

Chromotherapy is a non-invasive method that uses different colors to harmonize the body's energy and slowly mitigate the symptoms of PCOS. Red and orange colors in particular are frequently selected due to their association with the root and sacral chakras that are related to the reproductive and hormonal system. Besides, light-based therapies such as chromotherapy are gaining traction in terms of their potential to regulate the endocrine system and other related areas, yet the effectiveness of chromotherapy in PCOS still requires more rigorous clinical trials. Low-level laser therapy has been shown to improve ovarian function, reduce inflammation, and enhance menstrual regularity [42].

Magnetotherapy and PCOS

Magnetotherapy is a non-invasive treatment rooted in TCM principles, where small magnets are applied to specific acupressure points on the body (e.g., hands or feet). It is an effective treatment for PCOS that enhances uterine blood flow, relaxes muscles, and restores hormonal balance by applying magnets to specific body areas. It aims to improve energy balance, oxygen supply to the uterus, and reduce stress, addressing common PCOS symptoms. Research indicates that it may regulate the autonomic nervous system and affect reproductive functions by modifying neuropeptide and hormone secretion [43]. 

Diet and PCOS

Dietary therapy plays a major role in managing PCOS by addressing IR, hyperandrogenism, and metabolic disorders through low-glycemic index (GI), Mediterranean, and ketogenic diets. In addition to improving insulin sensitivity, these therapies help people lose weight, which improves reproductive outcomes like ovulation and regular menstruation. Low-GI diets that consist of whole grains, legumes, fiber-rich vegetables, and fruits help keep the blood glucose at a stable level, lower IR, and support ovulation in PCOS patients. Low-carbohydrate ketogenic diets can similarly improve fat burning and reduce fasting insulin, as well as free testosterone, and thus fertility can be better in obese women with PCOS [44]. The Mediterranean diet emphasizing anti-inflammatory foods like fruits, vegetables, olive oil, nuts, and lean proteins not only helps cardiovascular health but also lowers inflammation and balances hormones. High-protein diets have a better effect than balanced diets in terms of the reduction of fasting insulin, especially for a duration of 12 weeks, without having a significant effect on lipid profiles [45]. Vitamin D is a steroid hormone and is considered the main regulator of calcium metabolism and skeletal homeostasis, and it is also suggested to have significant metabolic and endocrine functions [46]. Omega-3 fatty acids, through their anti-inflammatory properties, may improve cardiovascular health and metabolic functions in patients with PCOS. Also, the utilization of inositol facilitates ovarian function and insulin signaling pathways, whereas probiotics assist in gut microbiota balance restoration, which in turn may alleviate hyperandrogenism as well as metabolic disturbances [47]. Herbal remedies show significant potential in managing PCOS symptoms, including infertility, hirsutism, hyperandrogenism, IR, and endometrial hyperplasia, with minimal side effects. Aloe vera enhances fertility by normalizing ovarian germ cells and increasing follicle production and LH secretion. Chamomile alleviates PCOS symptoms and supports follicle growth and LH secretion through its flavonoids and antioxidants. Vitex agnus-castus reduces hirsutism and testosterone levels, promoting regular menstrual cycles and increasing progesterone. Flaxseed lignans lower androgen levels and hirsutism, as shown in a study with PCOS patients receiving 30g daily [48]. In short, these herbs improve immunity, regulate menstrual cycles, and rectify hormonal imbalances with very few side effects, and thus, they can be considered as effective co-therapies in ​‍​‌‍​‍‌​‍​‌‍​‍‌PCOS.

Yoga and PCOS

Yoga can help those with PCOS achieve hormonal balance, reduce stress, improve metabolism, and regularize menstrual cycles. Through asanas, pranayama, and meditation, it can alleviate endocrine and nervous system dysfunctions, reducing anxiety, weight gain, and irregular periods. Additionally, yoga lowers urinary catecholamines and aldosterone, decreases serum testosterone and LH levels, and increases cortisol excretion, indicating positive changes in hormonal profiles [49]. Yoga meditation and practice significantly affect hormones and neurotransmitters such as corticotropin-releasing hormone, melatonin, and cortisol, among others. Additionally, yoga is associated with improvements in blood pressure, lipid levels, oxidative stress, coagulation, and immune status, potentially influencing metabolic health [50]. Few studies suggest that regular yoga practice (60 min of practice five days a week for a period of six months) helps in improving blood circulation to the pelvic viscera and improves reproductive functions by reducing stress and balancing the neurohormonal profile [51].

Future directions

Future research should focus on large-scale, multicenter RCTs to assess the effectiveness and safety of non-pharmacological therapies like hydrotherapy, acupuncture, yoga, dietary changes, and herbal remedies. Standardizing treatment protocols and outcome measures is essential for validating their benefits. Additionally, identifying molecular biomarkers and neuroendocrine pathways influenced by these therapies may lead to personalized integrative management strategies.

## Conclusions

A multimodal strategy combining evidence-based complementary therapies with standard treatments can enhance hormonal balance, metabolic function, mental well-being, and overall quality of life for women with PCOS. The future of PCOS treatment lies in a personalized, integrative, and preventive care model that addresses both physiological and psychosocial factors.
